# Portal Vein Thrombosis in Patients With Cirrhosis of the Liver: Prevalence and Risk Factors

**DOI:** 10.7759/cureus.50134

**Published:** 2023-12-07

**Authors:** Lokesh Koumar, Kuppusamy Senthamizhselvan, Deepak Barathi, Amogh Verma, Pallavi Rao, Jayachandran Selvaraj, Vivek Sanker

**Affiliations:** 1 Cardiology, Wolverhampton Heart and Lung Centre, New Cross Hospital, Wolverhampton, GBR; 2 Medical Gastroenterology, Jawaharlal Institute of Postgraduate Medical Education and Research, Puducherry, IND; 3 Radiodiagnosis, Jawaharlal Institute of Postgraduate Medical Education and Research, Puducherry, IND; 4 Medicine, Rama Medical College Hospital and Research Centre, Hapur, IND; 5 Internal Medicine, Dr. Rajendra Prasad Government Medical College, Kangra, IND; 6 General Internal Medicine, Jawaharlal Institute of Postgraduate Medical Education and Research, Puducherry, IND; 7 General Surgery, Noorul Islam Institute of Medical Science and Research Foundation (NIMS Medicity), Trivandrum, IND

**Keywords:** coagulopathy, anaemia, decompensated liver disease, cirrhosis, portal vein thrombosis

## Abstract

Introduction: Chronic liver disease very often culminates into cirrhosis and its associated complications. One of the serious complications is portal venous thrombosis, which can occur due to a variety of risk factors. One significant factor contributing to portal hypertension is portal vein thrombosis (PVT). In this study, we aimed to investigate the prevalence of PVT among patients with liver cirrhosis in a tertiary hospital and identify the factors associated with this complication.

Methodology: This was a cross-sectional observational study of 93 diagnosed liver cirrhosis patients treated at Jawaharlal Institute of Postgraduate Medical Education and Research (JIPMER) hospital in southern India between June 2020 and January 2021. A thorough evaluation of the clinical condition of the patients and associated comorbidities was done. The patients then underwent Doppler ultrasound/CECT/MRI to look for PVT and its extent. The collected data were analyzed using Statistical Product and Service Solutions (SPSS, version 24) (IBM SPSS Statistics for Windows, Armonk, NY). Comparison between two proportions was done using two two-tailed Z-test/Fisher's exact tests.

Results: Our study found a PVT prevalence of 17.2% in cirrhotic patients, with a higher prevalence of acute PVT than chronic PVT. Ascitic fluid infection, longer duration of cirrhosis, and increased cirrhosis severity were significantly associated with PVT development. We found no significant associations between PVT and gender, hypertension, smoking, diabetes, or the duration of alcohol intake.

Conclusion: This study highlights the importance of early screening for PVT using Doppler USG in all patients diagnosed with cirrhosis. Additionally, anticoagulation therapy for acute PVT may be considered in patients without bleeding risks.

## Introduction

Chronic liver disease (CLD) culminates in a debilitating condition known as liver cirrhosis, marking the end stage of progressive liver disorder [1]. Characterized by regenerative nodules ensnared by fibrous bands, this disease is frequently asymptomatic in its early stages, complicating its timely diagnosis and posing a significant challenge to secondary prevention [1]. It is during this period of compensated liver cirrhosis that the patient's struggle remains hidden while the disease silently progresses [1].

However, when cirrhosis advances to its decompensated form, the clinical landscape transforms dramatically [1]. Decompensated liver cirrhosis signifies liver cirrhosis, accompanied by the manifestation of symptoms or signs of portal hypertension [1-3]. These include the onset of complications such as ascites, gastrointestinal bleeding, elevated bilirubin levels, ascitic fluid infections, hepatic encephalopathy, and coagulation disorders, which result in a propensity for bleeding or thrombosis [1-3]. It is at this stage that patients, almost invariably, present to the hospital, illustrating the severe morbidity and high mortality that accompany decompensated liver cirrhosis [1-3].

Evaluating the global and Indian landscape of cirrhosis reveals a staggering burden of this ailment [3,4]. Sepanlou et al. conducted a study on the global prevalence of liver cirrhosis, spanning the years from 1990 to 2017 [3]. Their results underscored the extensive variation in the burden of liver cirrhosis across nations, ethnicities, genders, and time [3]. They reported a global prevalence exceeding 10 million, mostly affecting males [3]. In the same year, the global prevalence of compensated liver cirrhosis exceeded a staggering 112 million, with 58.8% of the cases involving males [3]. Similarly, Mukherjee et al., in their study conducted in India, noted that 1.2% of all hospital visits are attributed to CLD, including liver cirrhosis [2]. This condition also contributes to 2% of overall deaths in the country [2].

Patients with Budd-Chiari syndrome seem to be more affected by inherited and acquired thrombophilias than by portal vein thrombosis (PVT). However, more than one risk factor or cause was found in nearly half of the PVT patients [5]. PVT can be acute or chronic in clinical terms. While a timeframe is not specified, PVT is often deemed acute if symptoms appear less than 60 days before hospital evaluation [5]. The absence or negligible porto-portal collaterals on imaging, along with the lack of any signs of portal hypertension, such as splenomegaly and esophageal varices, are used to distinguish between acute and chronic PVT [5].

Acute PVT that is partially blocked may not cause any symptoms or vague ones. On the other hand, symptoms of decompensation of CLDs, such as variceal bleeding, worsening ascites, bloody diarrhea, peritonitis, intestinal ischemia, or portal cholangiopathy, can accompany an acute phase of a completely occluded thrombosis. Acute PVT may arise in a cirrhotic patient if there is a sudden clinical deterioration, such as the onset of bacterial peritonitis [6].

Distinguishing between compensated and decompensated liver cirrhosis is crucial to understanding the complex nature of this condition and its implications for patient care [1]. In the early stages, patients with compensated liver cirrhosis typically present with no symptoms or signs, making it challenging to diagnose due to their asymptomatic nature [1]. This discrepancy in clinical presentation contributes to the underestimation of the true prevalence of liver cirrhosis, as these patients rarely seek medical attention [1].

Cirrhosis exerts a significant global burden, with its prevalence varying widely based on various factors, including the severity of the disease [3]. PVT is one of the severe complications of liver cirrhosis. According to studies, in cases of compensated cirrhosis, the prevalence of PVT, a severe complication, has been reported to be as low as 1% [7]. However, when cirrhosis progresses, the prevalence of PVT escalates [8]. The intricacies of PVT are multifaceted and interconnected with a patient's age, the velocity of blood flow in the portal vein, underlying hypercoagulable states, the influence of medications, and the nature of the underlying liver pathologies [8]. Notably, PVT in cirrhosis can be a cause and a consequence of decompensation [7]. It can develop because of several aetiologies, including malignancy and non-malignant causes, further highlighting the complexity of this condition [9]. Clinical findings of PVT in cirrhosis vary from asymptomatic to life-threatening conditions [10]. Partial PVT, which is now often detected by routine ultrasonography or computed tomography, might be associated with a few symptoms [10]. However, complete PVT may present as abdominal or lumbar pain with sudden onset or progression over a few days [10].

In a study conducted between 2005 and 2017 at a gastrointestinal ward in India, a total of 16,902 patients were admitted [4]. The focus of this study was on patients with liver cirrhosis, comprising 4,331 individuals [4]. Notably, 63.3% of these cases were attributed to alcohol-related liver cirrhosis, affecting 2,742 patients [4]. Additionally, 19.8% of the cases were associated with liver cirrhosis related to viral hepatitis, impacting 858 patients [4]. Another 16.9% of the cases involving 731 patients were identified as liver cirrhosis attributed to non-alcoholic and non-viral causes [4]. In light of the significant burden of liver cirrhosis and its associated complications, particularly PVT, this study aims to contribute to our understanding of the prevalence and risk factors of PVT in cirrhotic patients.

## Materials and methods

Study design

This was a cross-sectional observational study of 139 diagnosed liver cirrhosis patients treated at Jawaharlal Institute of Postgraduate Medical Education and Research (JIPMER) hospital in southern India between June 2020 and January 2021. The study was approved by the hospital's research and ethics committee (JIP/IEC/2020/188).

Inclusion criteria

All patients aged more than 18 years with liver cirrhosis who were admitted or who attended the outpatient department at JIPMER hospital are included in the study. Additionally, patients who satisfied clinical and imaging criteria for liver cirrhosis as per the study protocol were included in the study. Baseline tests included hemograms, liver function tests (LFT), prothrombin time, international normalized ratio (PT/INR), and serum creatinine. The severity of liver cirrhosis was graded based on Child-Pugh scoring.

Exclusion criteria

Patients with known coagulation disorders, on anticoagulant therapy, post-splenectomy, or suffering from hepatocellular carcinoma were excluded from the study.

Data collection and statistical analysis

**Figure 1:**
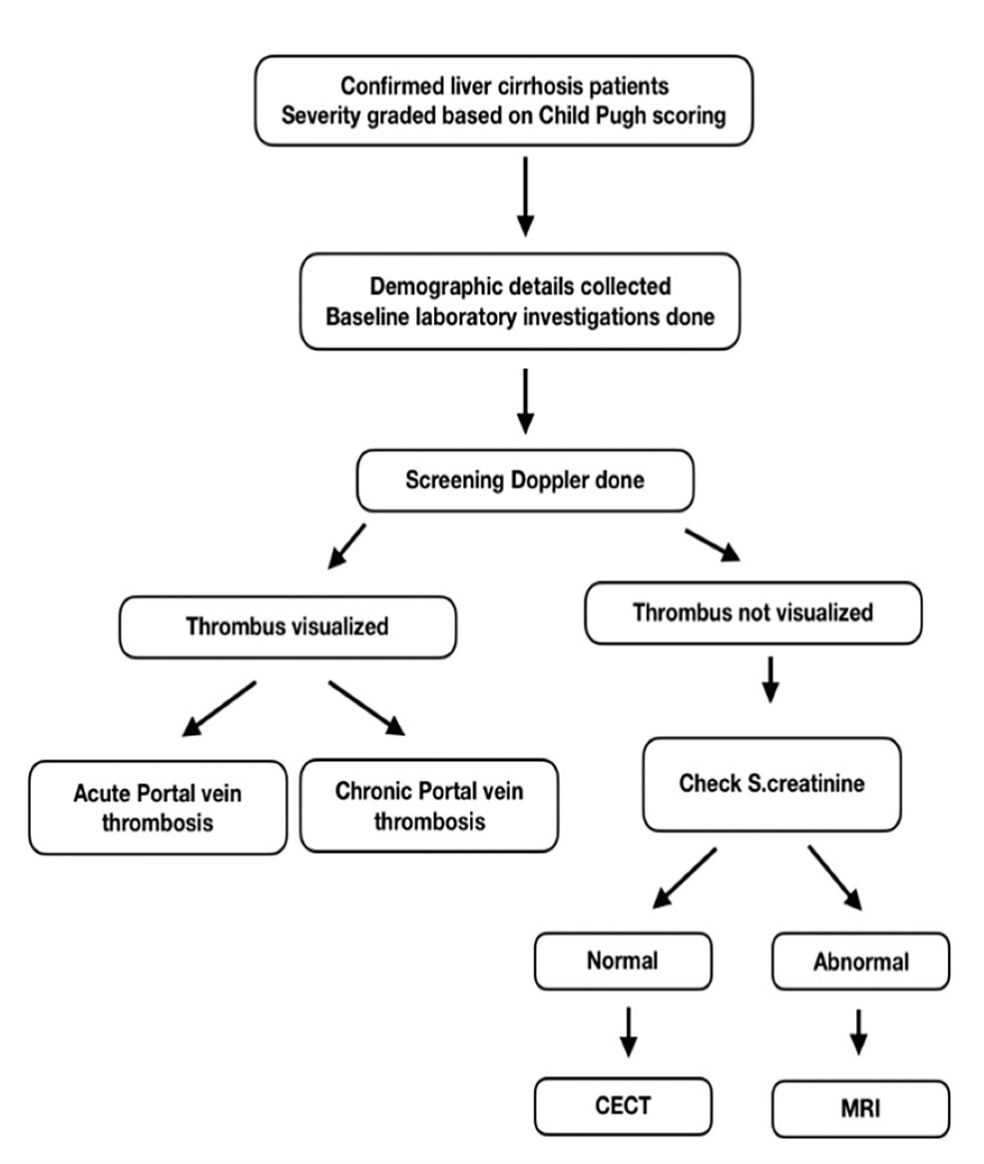
Flow chart depicting the working protocol

Patients with confirmed liver cirrhosis underwent a Doppler scan to look for PVT. When PVT was identified, its extent was noted. When Doppler was inconclusive, a contrast-enhanced computed tomography (CECT) scan was done to confirm the presence and extent of PVT. When the patient’s recent (<2 weeks) serum creatinine was more than or equal to 1.4 mg/dL, a CT scan was not performed, and magnetic resonance imaging (MRI) imaging was done. Similarly, where the Doppler did not show any evidence of PVT, a CT scan was not performed (Figure 1).

The duration of follow-up was limited to the duration of the patient’s hospital stay. The findings of the Doppler and CT scan were shared with the treating physician, but no attempt was made to influence the treatment thenceforth. The collected data were analyzed using Statistical Product and Service Solutions (SPSS, version 24) (IBM SPSS Statistics for Windows, Armonk, NY). Comparison between two proportions was done using two two-tailed Z-test/Fisher's exact tests. A p value < 0.05 is considered significant.

## Results

A total of 93 patients were found eligible to participate in the study. Eighty-six (92.5%) of the patients were males, whereas seven (7.5%) patients were females, with a male-to-female ratio of 12.3:1.

Seventy-nine (84.9%) of the study patients were alcoholics. Out of these, 27 (34.2%) were alcoholics for less than five years, 35 (44.3%) patients were alcoholics for 6-10 years, and 17 (21.5%) patients were known alcoholics for 11-20 years. Of the 93 patients, 20 (21.5%) were smokers. The duration of smoking was less than five years in the case of three (15%) patients, 6-10 years for six (30%) patients, 11-20 years for 10 (50%) patients, and more than 20 years for one (5%) patient. Thus, the majority were chronic smokers - 17 (85%). Only four patients (4.4%) out of the total study population were positive for hepatitis B infection.

Out of the total of 93 patients, 41 (44.09%) were known to have had liver cirrhosis for less than a year, 50 (53.76%) suffered from cirrhosis for one to five years, and only two (2.15%) suffered from cirrhosis for more than five years. Sixty-seven (72.04%, i.e., the majority of the cirrhotic patients) belonged to Child-Pugh category C, and 15 (22.4%) among them were found to have PVT. Twenty-four (25.81%) of the cirrhotic patients belonged to category B, but only one among them showed PVT on imaging. Only two of the study population (2.15%) belonged to category A, and none of them were found to have PVT. Among the study population, 24 (26%) had clinical/laboratory features suggestive of ascitic fluid infection, and 11(11.8%) patients had the presence of esophageal varices. The distribution of the study population based on different variables and risk factors is shown in Table 1.

**Table 1:** Distribution of the study population based on different variables and risk factors HBV: Hepatitis B Virus, PVT: Portal Vein Thrombosis The data have been represented as N with its corresponding percentage (%).

Category	Observation		No. of patients	% of patients
Gender	Male		86/93	92.5%
	Female		7/93	7.5%
Alcoholics	Alcoholic		79/93	84.9%
	Duration of alcoholism	<5yr	27/79	34.2%
		6-10years	35/79	44.3%
		11-20years	17/79	21.5%
	Nonalcoholic		14/93	15.1%
Smoking	Smoker		20/93	21.5%
	Duration of smoking	<5 years	3/20	15%
		6-10years	6/20	30%
		11-20years	10/20	50%
		>20 years	1/20	5%
	Nonsmoker		73/93	78.5%
HBV	Positive		4/93	4.4%
	Negative		89/93	95.6%
Cirrhosis	Duration of cirrhosis	<1 years	41/93	44.09%
		1-5 years	50/93	53.76%
		>5 years	2/93	2.15%
	Child-Pugh Category	A	2/93	2.15%
		B	24/93	25.81%
		C	67/93	72.04%
Ascitic infection	Positive		24/93	26%
	Negative		69/93	74%
Esophageal varices	Present		11/93	11.83%
	Absent		82/93	88.17%
PVT	Acute		14/93	15%
	Chronic		2/93	2%
	None		67/93	83%

Among the study participants,15% were found to have acute PVT, and 2% were diagnosed to have chronic PVT. PVT was most common among cirrhotic patients 15/67 (22.4%) belonging to Child-Pugh category C. It was observed that PVT developed more frequently in patients (14/91) with cirrhosis of ≤5 years duration in comparison to those (2/2) with cirrhosis of >5 years duration, and this was statistically significant. Additionally, a statistically significant number of severe cirrhosis patients developed PVT in comparison with patients with mild-to-moderate cirrhosis.

**Figure 2:**
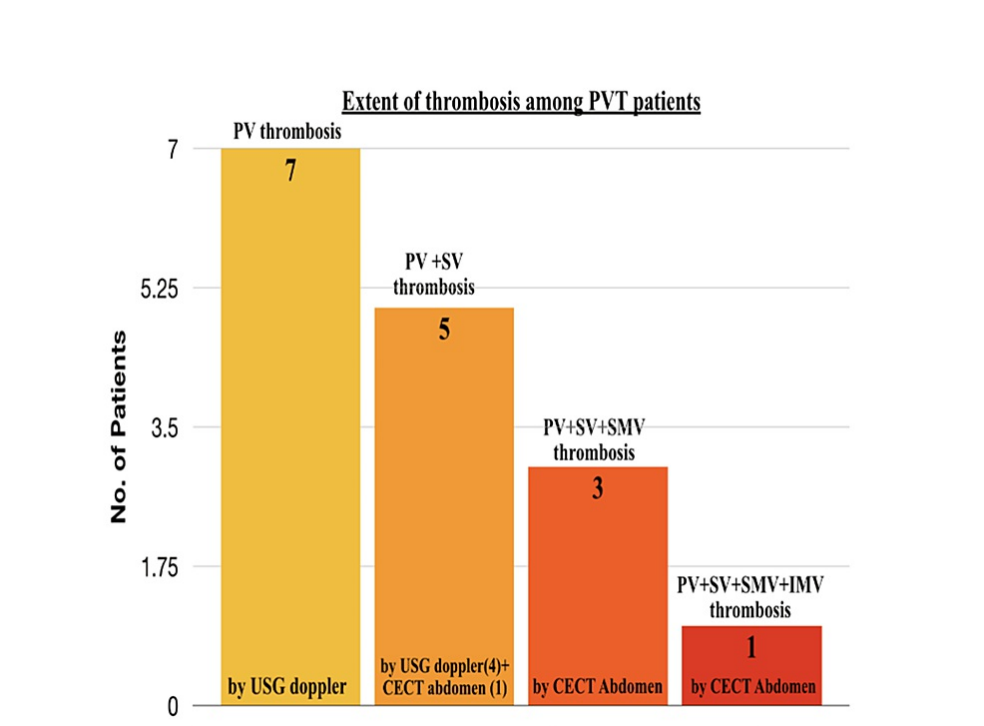
Number of patients who are found to have thrombosis in different veins using imaging by USG Doppler or CECT PV: Portal Vein, SV: Splenic Vein, SMV: Superior Mesenteric Vein, IMV: Inferior Mesenteric Vein, CECT: Contrast-Enhanced Computed Tomography, USG: Ultrasonography, PVT: Portal Vein Thrombosis

Twenty-four participants were found to have ascitic fluid infection, out of which 10 developed PVT, which was also statistically significant. Gender did not appear to play a role in the development of PVT. The difference between the proportion of PVT in patients with a duration of alcoholism of less than five years, and more than five years was not big enough to be statistically significant. Out of 16 patients, seven patients were diagnosed with PVT by USG Doppler. Four patients had portal and splenic vein thrombosis detected by ultrasonogram (USG) Doppler. One patient had portal and splenic vein thrombosis confirmed by contrast-enhanced computed tomography (CECT) abdomen. Three patients had portal, splenic, and superior mesenteric vein extension detected by the CECT abdomen, and one PVT with splenic, superior mesenteric vein and inferior mesenteric vein extension was confirmed by the CECT abdomen (Figure 2). The development of PVT and its association with various variables is shown in Table 2.

**Table 2:** Number of patients (% of patients) found to have portal vein thrombosis with respect to different variables and risk factors PVT: Portal Vein Thrombosis, DM: Diabetes Mellitus, HT: Hypertension, S: Significant, NS: Not Significant

Category	Findings	PVT in a number of patients (% of patients)		P value
		Present	Absent	
Gender	Male	13 (15.1%)	73 (84.9%)	0.0515 (NS)
	Female	3 (42.9%)	4 (57.1%)	
Duration of cirrhosis	Total	16 (17.2%)	77 (82.7%)	0.0017 (S)
	≤5 years	14 (15.4%)	77 (84.6%)	
	>5 years	2 (100%)	0 (0%)	
Severity of cirrhosis	mild/mod	1(3.8%)	25 (96.2%)	0.0335 (S)
	Severe	15 (22.4%)	52 (77.6%)	
Child-Pugh category	A (n=2)	0/2 (0%)	2/2 (100%)	0.059 (NS)
	B (n=24)	1/24 (4.2%)	23/24 (95.8%)	
	C (n=67	15/67 (22.4%)	52/67 (77.6%)	
DM	Yes	1 (6.3%)	20 (26%)	0.108 (NS)
	No	15 (93.7%)	57 (74%)	
HT	Yes	3 (18.8%)	23 (30%)	0.541 (NS)
	No	13 (81.2%)	54 (70%)	
Smoking	Yes	5 (31.3%)	15 (19.5%)	0.321 (NS)
	No	11 (68.7%)	62 (80.5%)	
Alcoholism	Yes	12 (75%)	67 (87%)	0.252 (NS)
	No	4 (25%)	10 (13%)	
Duration of alcoholism	≤5 years	8 (20%)	32 (80%)	0.5439 (NS)
	> 5 years	8 (15.1%)	45 (84.9 %)	
HBV infection	Yes	1 (6.3%)	3 (3.9%)	0.53 (NS)
	No	15 (93.7%)	74 (96.1%)	
Ascitic infection	Yes	10 (62.5%)	14 (18.2%)	0.0002 (S)
	No	6 (37.5%)	63 (81.8%)	
Esophageal varices	Yes	7 (43.8%)	4 (5.2%)	0.0002 (S)
	No	9 (56.2%)	73 (94.8%)	
Hepatic encephalopathy	Yes	1 (6.3%)	10 (13%)	0.0002 (S)
	No	15 (93.7%)	67 (87%)	
Albumin levels	Normal	1 (25%)	3 (75%)	0.6793
	Reduced	15 (16.9%)	74 (83.1%)	

## Discussion

Decompensated CLD (DCLD) refers to advanced stages of chronic liver disease where the liver can no longer perform its vital functions adequately [11]. Common causes of DCLD include viral hepatitis, alcohol-related liver disease, non-alcoholic fatty liver disease (NAFLD), and cirrhosis due to various factors [11]. Alcoholic liver damage can arise from daily alcohol consumption of 30-50 g for longer than five years. Ninety percent of patients who use more than 60 g of alcohol per day have a high probability of developing steatosis, and 30% of those who consume more than 40 g of alcohol over an extended period of time have a higher risk of developing cirrhosis [12]. In DCLD, extensive liver damage leads to loss of liver function, resulting in symptoms such as ascites, jaundice, hepatic encephalopathy, and variceal bleeding [11].

PVT involves blood clot formation in the portal vein [3]. Key factors contributing to PVT are hypercoagulable states, liver cirrhosis, abdominal infections, surgery/trauma, vascular abnormalities, and prothrombotic conditions [3]. Understanding these causes is essential for diagnosis and tailored treatment of both DCLD and PVT [3].

In this study, we aimed to investigate the prevalence of PVT among patients with liver cirrhosis and identify the factors associated with this complication. Our findings provide important insights into the occurrence of PVT in cirrhotic patients and shed light on the potential risk factors contributing to its development.

Prevalence of PVT

Our study revealed that the prevalence of PVT among patients with cirrhosis was 17.2%. This finding underscores the significance of PVT as a relatively common complication in this patient population. Notably, we observed that 15% of the cases presented as acute PVT, while 2.2% were identified as chronic PVT. Most PVT cases were located in the main trunk of the portal vein, with extensions to the splenic vein in 56.3% and the superior mesenteric vein in 25% of cases.

Our results align with previous studies reporting varying prevalence rates of PVT in cirrhotic patients, ranging from 0.6% to 26% [4,5,13,14]. The wide prevalence range may be attributed to several factors, including cirrhosis severity, age, portal blood flow velocity, hypercoagulable states, underlying liver pathologies, and other factors [8,15-17]. It is important to acknowledge that the prevalence of PVT is influenced by these variables and may vary among different patient populations. The recognition of PVT as a relatively common complication in cirrhotic patients underscores the need for vigilant monitoring and preventive measures.

Association with the severity of cirrhosis

Our study demonstrated a significant association between the severity of liver cirrhosis and the development of PVT. Specifically, patients classified as Child-Pugh category C exhibited a significantly higher risk of developing PVT. The odds of developing PVT were 4.7 times higher in patients with Child-Pugh category C compared to those with Child-Pugh category B. This finding is consistent with previous research by Zhang et al., supporting the notion that the severity of cirrhosis is a relevant factor in the development of PVT [3,18,19]. This emphasizes the clinical importance of assessing cirrhosis severity when evaluating the risk of PVT in cirrhotic patients.

Ascitic fluid infection and its relationship with PVT

Our study delved into the connection between ascitic fluid infection, both clinically and through laboratory evidence, and the onset of PVT. Notably, our findings demonstrated a robust correlation, indicating a heightened PVT risk in patients with signs of ascitic fluid infection. Escherichia coli emerged as the most frequently identified organism in cases of ascitic fluid infection. Additionally, the presence of portal endotoxemia associated with ascitic fluid infection was suggested to elevate thrombosis risk in cirrhotic patients, a relationship consistent with prior studies [20]. This insight is pivotal for comprehensively evaluating thrombotic risk factors in cirrhotic patients. In end-stage liver disease, infection and inflammation can induce alterations in portal pressure, potentially contributing to PVT development - a trend observed in other studies as well [6,21].

Duration of cirrhosis

We found that a duration of cirrhosis of five years or longer was associated with the occurrence of PVT in our study. This observation is in line with a study by Trifan et al., which reported an increasing cumulative incidence of cirrhosis over the years following the initial diagnosis [22]. However, the assessment of the duration of cirrhosis at the time of presentation is challenging due to the largely asymptomatic nature of compensated cirrhosis. Therefore, further research is needed to establish the independent role of cirrhosis duration as a risk factor for PVT.

Hypoalbuminemia and its relationship with PVT

Our study identified a potential association between hypoalbuminemia and PVT. A substantial proportion of patients with hypoalbuminemia also had PVT, suggesting clinical significance. Hypoalbuminemia, which can result from albumin loss in ascitic fluid and low levels due to acute-phase reactions in decompensated liver disease and ascitic fluid infection, has been recognized as an independent risk factor for venous thromboembolism [23-25]. Although this trend was observed in our study, the statistical significance was not reached, likely due to the relatively small sample size.

Factors not associated with PVT

Our study found no significant associations between PVT and gender, hypertension, smoking, diabetes, or the duration of alcohol intake. Notably, the representation of female patients in our study was limited, making it difficult to draw definitive conclusions regarding the prevalence of PVT in females.

Anticoagulation and follow-up

Among the patients with acute PVT in our study, some received anticoagulation treatment following a risk assessment for bleeding conducted by their treating physicians. However, our study did not include follow-up data to assess the recanalization of PVT in these patients.

Strengths and limitations

The strengths of this study lie in being the first in South India to investigate the prevalence of PVT in cirrhotic patients and its associated risk factors. Moreover, our study included non-malignant PVT and considered various risk factors beyond thrombophilic disorders.

Nonetheless, this study faced certain limitations. The sample size was constrained due to difficulties during the COVID-19 pandemic, limiting our ability to reach more participants. Additionally, the study had relatively few female participants, which hindered an accurate determination of the prevalence of PVT in females. Finally, the extent of the thrombus could have been better characterized using CECT scans, but this was avoided due to the potential risks associated with contrast and radiation exposure.

Future perspectives

Based on our findings, we propose several future perspectives in managing PVT in cirrhotic patients. Initially, we recommend regular screening for PVT using Doppler ultrasonography in all patients diagnosed with cirrhosis. Furthermore, considering anticoagulation for acute PVT in patients without bleeding risks should be explored, but further research is needed to establish clear guidelines for practice.

Our study highlights the prevalence of PVT in cirrhotic patients and its association with several key factors, including the severity of cirrhosis, ascitic fluid infection, and cirrhosis duration. Understanding these risk factors is crucial in determining the treatment outcome and, hence, the prognosis of these patients.

## Conclusions

PVT is a significant and treatable complication in patients with liver cirrhosis. This study aimed to determine the prevalence of PVT in cirrhotic patients and identify associated risk factors. A total of 93 patients diagnosed with liver cirrhosis were included in the study, and Doppler USG was performed to screen for PVT. Our study found a PVT prevalence of 17.2% in cirrhotic patients, with a higher prevalence of acute PVT than chronic PVT. Ascitic fluid infection, longer duration of cirrhosis, history of hepatic encephalopathy, oesophageal varices, and increased cirrhosis severity were significantly associated with PVT development. While hypoalbuminemia was observed in PVT cases, it did not show a statistically significant association in this study, possibly due to sample size limitations. These findings highlight the importance of early screening for PVT using Doppler USG in all patients diagnosed with cirrhosis. Additionally, anticoagulation therapy for acute PVT may be considered in patients without bleeding risks, but further research is needed to support this approach.

## Appendices

Appendix - 1

Data Collection Proforma

Patient Name:                                         Age:                 Sex:                                                              Hospital No.

**Table 3:** Data collection proforma GI: Gastrointestinal, Hb: Hemoglobin, WBC: White Blood Count, PS: Peripheral Smear, AST: Aspartate Aminotransferase, ALT: Alanine Transaminase, GGT: Gamma-Glutamyl Transferase, PT: Prothrombin Time, MRI: Magnetic Resonance Imaging, IBD: Inflammatory Bowel Disease

DEMOGRAPHIC DETAILS				
Socioeconomic status				
VARIABLES				
H/O smoking				
H/O alcohol consumption				
H/O viral infection				
Duration of liver cirrhosis				
Severity of cirrhosis (Child-Pugh score)				
H/O spontaneous bacterial peritonitis	Yes/No			
-If yes – cultured organism				
H/O Variceal / Upper GI bleed	Yes/No			
-If yes – h/o any sclerotherapy/ banding				
H/O hepatic encephalopathy				
INVESTIGATION				
Complete hemogram with peripheral smear		Hb		
		WBC count		
		Differential count		
		Platelet		
		PS		
Liver function test		Total bilirubin		
		Direct bilirubin		
		Indirect bilirubin		
		AST		
		ALT		
		ALP		
		GGT		
		S. total protein		
		S. albumin		
Renal function test		S. urea		
		S. creatinine		
		S. Na		
		S. K+		
		S. Cl-		

PT/INR				
RADIOLOGICAL INVESTIGATION				
USG abdomen				
USG Doppler				
CECT abdomen				
MRI				
FACTORS		Diabetes mellitus		
		Systemic hypertension		
		Malignancy		
		IBD		
		H/o splenectomy		
